# Overexpression of RPN2 promotes osteogenic differentiation of hBMSCs through the JAK/STAT3 pathway

**DOI:** 10.1002/2211-5463.12766

**Published:** 2019-12-14

**Authors:** Ling Ni, Jianhua Yu, Xueqiong Gui, Zhonghua Lu, Xiwen Wang, Hongyan Guo, Ying Zhou

**Affiliations:** ^1^ Department of Geriatrics Yangpu District Shidong Hospital Shanghai China

**Keywords:** bone mesenchymal stem cell, hBMSC, JAK/STAT3, osteogenic differentiation, RPN2

## Abstract

Osteoporosis is characterized by decreased bone mass and degenerating bone structure, which cause severe bone fragility and increase the risk for fractures. Human bone mesenchymal stem cells (hBMSCs) differentiate into osteoblasts through osteogenesis, and disturbances in the balance between bone generation and degeneration underlie the pathogenesis of senile osteoporosis. The highly conserved glycoprotein Ribophorin II (RPN2) is involved in multiple biological reactions, but the role of RPN2 in the osteogenic differentiation of hBMSCs and their molecular etiology is incompletely understood. Here, we show that RPN2 expression is up‐regulated in hBMSCs during osteogenic differentiation. *In vitro* assays revealed that silencing of RPN2 inhibited hBMSC differentiation into osteoblasts. Moreover, RPN2 overexpression enhanced the expression of linked genes and resulted in high alkaline phosphatase activity. Our results suggest that RPN2 targets Janus kinase 1 (JAK1), and RPN2 overexpression was observed to induce JAK1 ubiquitination. Depletion of JAK1 facilitated osteogenic differentiation of RPN2‐silenced hBMSCs. Moreover, western blot analysis revealed that RPN2 silencing suppressed the stimulation and nuclear translocation of the downstream signal transducer and activator of transcription 3 sensor; this could be reversed via RPN2 overexpression. This research sheds light on an innovative molecular mechanism that is associated with hBMSC differentiation into osteoblasts and may facilitate bone anabolism through RPN2.

## Graphical abstract

Ribophorin II overexpression induces Janus kinase 1 ubiquitination and degradation, and subsequently inhibits phosphorylation and nuclear localization of the downstream signal transducer and activator of transcription 3 sensor. Nuclear signal transducer and activator of transcription 3 facilitates expression of osteogenic differentiation‐specific genes. However, this phenomenon could be reversed via Ribophorin II silencing.

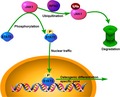

**Abbreviations**
ALP: alkaline phosphatase
BMP: bone morphogenetic protein
DAPI: 4',6-diamidine-2'-phenylindole dihydrochloride
CdCl_2_: cadmium chloride
hAS: human allogeneic serum
hBMSC: human bone mesenchymal stem cell
JAK1: Janus kinase 1
NC: negative control
NCi: negative control siRNA
PFA: paraformaldehyde
qRT‐PCR: quantitative RT‐PCR
RPN2: ribophorin II
SD: standard deviation
STAT3: signal transducers and activators of transcription
WB: western blotting

Osteoporosis is characterized by decreased bone mass and degenerating bone structure, which cause severe bone fragility and increase the risk for fractures 1, 2. This illness is categorized as primary type 2 (also called senile osteoporosis), type 1 (also named postmenopausal osteoporosis), or secondary (steroid‐ or glucocorticoid‐triggered osteoporosis, among others). Osteoporosis is caused by an imbalance between osteoblast‐modulated bone generation and osteoblast‐modulated bone degeneration in the marrow microenvironment 3, 4. Bone mesenchymal stem cells (BMSCs) belong to pleiotropic cells that have the ability to differentiate into osteoblasts, and osteogenesis is a set of complex reactions that regulates this differentiation process 5, 6. In terms of human BMSCs (hBMSCs), the production of distinct phenotypes is regulated via various growth factors and signaling pathways, which are related to a prevalent precursor located in fat, bone marrow and certain tissues 7.

The highly conserved glycoprotein Ribophorin II (RPN2) exists only in rough endoplasmic reticulum membranes and contributes to the translocation of secretory proteins and maintenance of the intrinsic uniqueness of the rough endoplasmic reticulum 8, 9. Early studies have indicated that the oligosaccharyltransferase complex contains RPN2 protein and conjugates oligosaccharides and asparagine residues in the N‐X‐S/T common motif of the polypeptide strand 10, 11. In addition, previous studies have demonstrated that RPN2 depletion suppresses the multiplication of tumor cells in osteosarcoma 12 and non‐small‐cell lung cancer 13, 14. Silencing of RPN2 was observed to inhibit malignant breast tumors by inhibiting glycosylation of CD63 15, which is a cell surface glycoprotein controlling cell mobility, invasiveness and metastasis 16. Similarly, the down‐regulation of RPN2 triggers docetaxel‐dependent programmed cell death and inhibits cell proliferation in the case of breast carcinoma by inhibiting N‐glycosylation of the P‐glycoprotein, as well as disrupting its membrane localization 17. However, its role in the osteogenic differentiation of hBMSCs in senile osteoporosis has not been probed.

The Janus kinase/signal transducers and activators of transcription (JAK/STAT) axis is an inflammatory‐associated pathway that is activated after receptor ligation 18, 19. It mediates the signaling from cytokine receptors to the nucleus. JAKs are activated after stimulation by cytokine receptors, resulting in phosphorylation and activation of the STAT family of transcription factors. Levy *et al*. 20 showed that elimination of the STAT pathway remarkably promoted bone morphogenetic protein‐triggered osteogenic differentiation of hBMSCs. Huang *et al*. 21 demonstrated that synergistic osteogenic function between bone morphogenetic protein 9 and growth hormone is noticeably blunted by JAK/STAT repressors. However, the contribution of the JAK1 and STAT3 pathways to hBMSC osteogenic differentiation remains to be studied.

## Materials and methods

### Cell cultivation and differentiation

Telomerase‐immortalized hBMSCs were acquired from primary hBMSCs 22 and were cultured in Dulbecco's modified Eagle's medium. Differentiation stimulation was carried out based on previous approaches 23, 24. Osteoblasts were recognized by the examination of alkaline phosphatase (ALP) activity after culturing 25, matrix mineralization was assessed by Alizarin Red staining, and cell proliferation and survival were evaluated by alamarBlue assay (provided by AbD Serotec).

### Von Kossa staining and Alizarin Red S staining

Culture medium was removed, and cells were washed twice with PBS before fixation in 4% paraformaldehyde (PFA) for 25 min. After PFA was removed, cells were washed twice with distilled water and allowed to air‐dry.

To detect calcium, we stained cells with 1% Alizarin Red S (Sigma‐Aldrich, St. Louis, MO, USA) adjusted to pH 6.3 by ammonium hydroxide (Wako‐Junyaku, Tokyo, Japan) for 15 min at 37 °C. Cells were washed twice with distilled water and incubated in 1 mL of 1% HCl in 70% ethanol for 1 h at 4 °C, as previously reported 26. Solutions (10 µL) were then applied to wells of a 96‐well assay plate (Sumilon, Tokyo, Japan), and *A* was determined at 450 nm (*A*_450 nm_) by Microplate Reader Model 680 (Bio‐Rad, Hercules, CA, USA).

To detect calcium phosphate and calcium carbonate, we stained fixed cells with 5% silver nitrate solution (Sigma‐Aldrich) dissolved in distilled water for 1 h under UV light, before washing twice with distilled water. To analyze von Kossa staining in a time course, we took photographs at each time point and analyzed digital images by imagej software (National Cancer Institute, Bethesda, MD, USA). All images were converted from color to grayscale to measure relative density and brightness.

### Transfection

An overexpressing vector pcDNA3‐RPN2 was constructed. Silencing of RPN2 and JAK1 was conducted by RPN2 and JAK1 siRNA transfection. Transfection was carried out by a vector using Lipofectamine 2000 (Invitrogen, Carlsbad, CA, USA) at day 15 poststimulation.

### Western blotting

hBMSC lysates were treated with radioimmunoprecipitation assay buffer, and then proteins were quantified using a bicinchoninic acid assay kit. Subsequently, proteins that were isolated on gel using 10% SDS/PAGE were moved to poly(vinylidene fluoride) membranes. Sites without membrane binding underwent blocking with 5% BSA, which was dissolved in PBS, including Tween 20 (PBST) for 1 h. The membrane protein was incubated overnight with primary antibodies at 4 °C, followed by incubation with secondary antibodies. Protein bands were evaluated, whereas specific gray values were assessed with a C‐DiGit Blot Scanner (LI‐COR Biosciences, Lincoln, NE, USA).

### RNA isolation and quantitative RT‐PCR

Total RNA was isolated from cells according to standard approaches. In short, quantitative RT‐PCR (qRT‐PCR) was carried out in a 20‐μL system with a SYBR Green Kit. Amplification processes were as follows: pre‐denaturation at 95 °C for 10 min, followed by denaturation at 95 °C for 15 s, annealing at 60 °C for 30 s and extension at 72 °C for 30 s for a total of 40 cycles. Quantification was carried out based on a $2^{-\Delta \Delta C_{T}}$ approach via glyceraldehyde‐3‐phosphate dehydrogenase normalization, which was associated with a calibrator (mean of controls).

### Immunofluorescence assays

Cells were cultivated in 24‐well plates with cover slides in osteogenic differentiation. Cells were fixed with 4% PFA and permeabilized with PBST at 25 °C for 15 min. Cells underwent 1‐h blocking with 0.4% BSA in PBST at 37 °C, before 1‐h incubation with STAT3 antibodies, which were deliquated with PBST including 0.2% BSA at 37 °C. Cells were incubated for 1 h with goat anti‐rabbit IgG, with TRITC labels deliquated in BSA (0.2%) in PBST at 37 °C, subsequent to 1‐h PBST washing. Cells underwent 1‐h PBST washing before nuclear staining with 4′,6‐diamidine‐2′‐phenylindole dihydrochloride (DAPI). STAT3 staining of cells was assessed with an Olympus LSCMFV500 confocal laser scanning fluorescence microscope (Olympus, Tokyo, Japan).

### Statistical analysis

Data were described as mean ± standard deviation (SD). Differences were assessed by ANOVA or two‐tailed Student's *t*‐test. A *P*‐value <0.05 was regarded as significant.

## Results

### RPN2 expression was up‐regulated in hBMSC osteogenic differentiation

**Figure 1:**
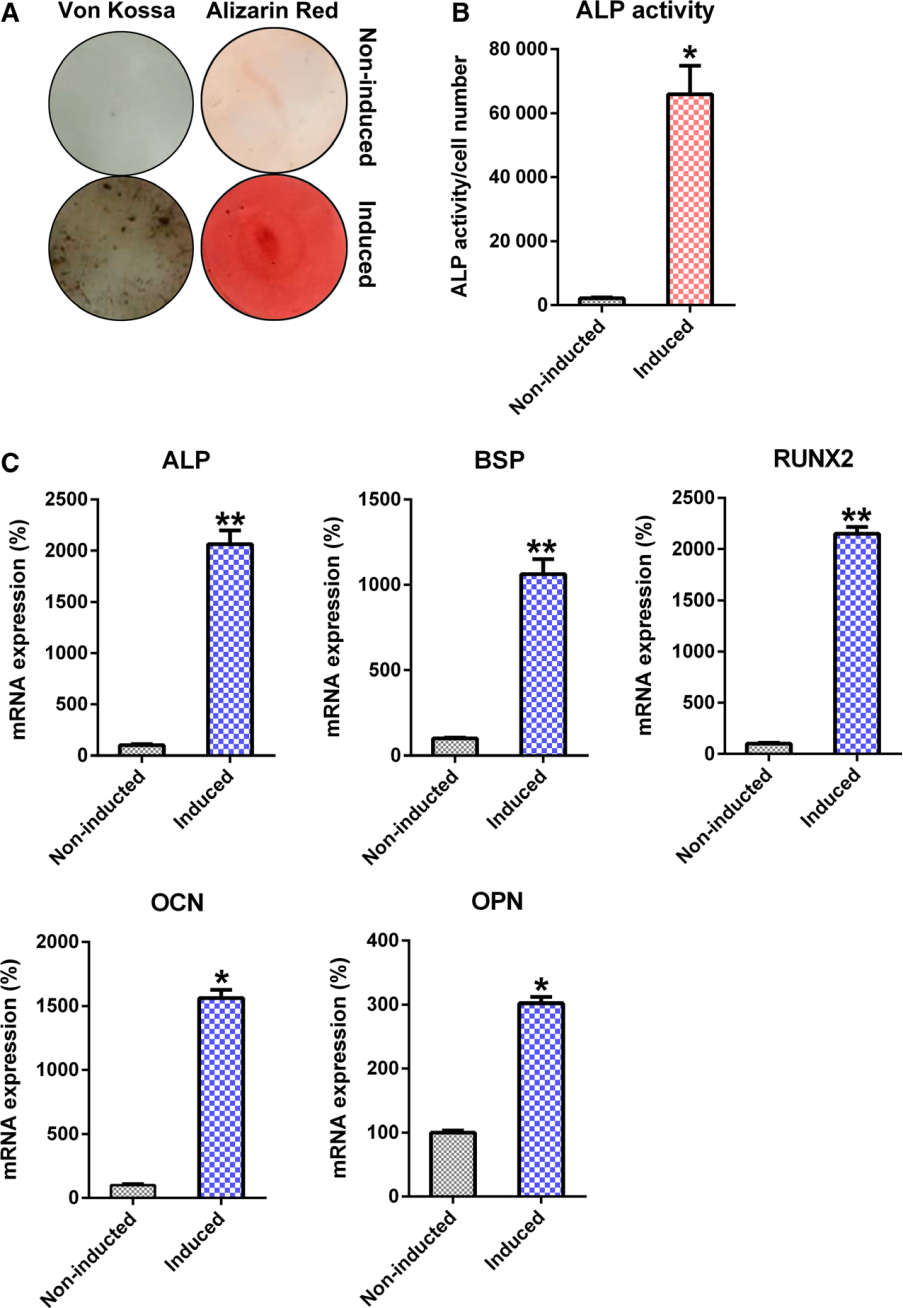
Osteoblastic differentiation of hBMSCs. Osteogenic differentiation was triggered in hBMSCs. (A) Alizarin Red staining and von Kossa staining results at the 14th day subsequent to stimulation. (B) ALP function in osteoblastic differentiation. (C) qRT‐PCR of OCN, OPN, RUNX2, BSP and ALP, and glyceraldehyde‐3‐phosphate dehydrogenase normalization at certain time points after stimulation. These tests were repeated three times. Differences were assessed by two‐tailed Student's *t*‐test. Error bars represent SD. *P < 0.05, **P < 0.01 versus noninduced groups.

hBMSCs isolated from four different donors, in passages 5–6, positive for CD166 and CD105 (98.9 ± 1.2% and 99.3 ± 1.0%, respectively) and negative for CD45 and CD34 (0.2 ± 0.2% and 0.5 ± 0.1%, respectively) 27, were used in this study. hBMSC osteogenic differentiation was stimulated via incubation in osteoblast‐stimulating medium. The successful osteogenic differentiation of hBMSCs was evidenced by ALP activity and relevant gene expression. An increased ALP activity was a typical osteoblastic phenotype observed in the induced group compared with the noninduced group (Fig. 1A,B). At day 14 after incubation, expression of genes related to osteoblastic differentiation (*ALP*, *BSP*, *RUNX2*, *OCN* and *OPN*) was up‐regulated (Fig. 1C).

**Figure 2:**
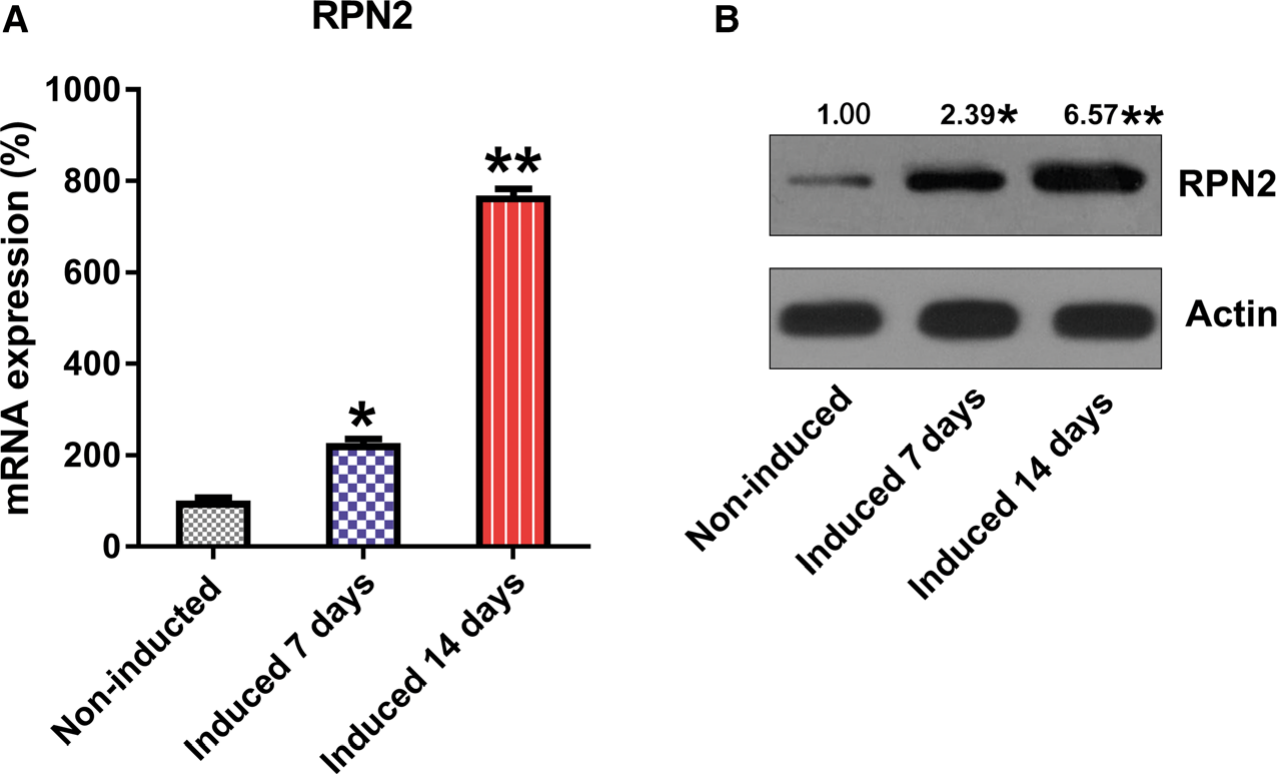
Expression of RPN2 during osteoblastic differentiation of hBMSCs. (A) qRT‐PCR and (B) WB detected the expression of RPN2 in osteoblastic differentiation. These tests were repeated three times. Differences were assessed by two‐tailed Student's *t*‐test. Error bars represent SD. *P < 0.05 versus noninduced groups; **P < 0.01.

Furthermore, qRT‐PCR and western blotting (WB) showed up‐regulated expression of RPN2 at day 14 after induction at both mRNA and protein levels, apart from stimulated osteogenic differentiation of hBMSCs (Fig. 2A,B).

### Regulation of RPN2 expression mediates the osteogenic differentiation process of hBMSCs

**Figure 3:**
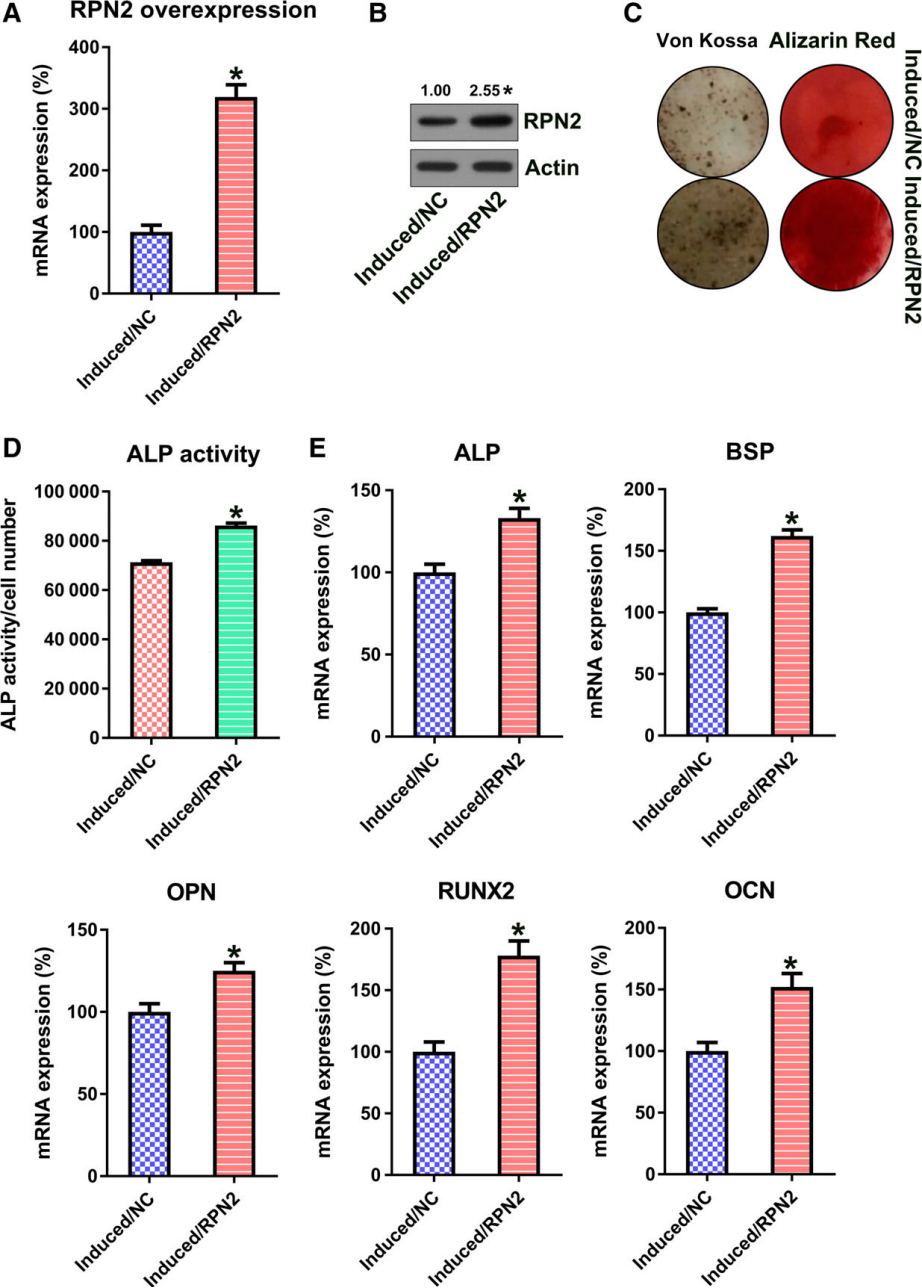
RPN2 overexpression promoted osteoblastic differentiation of hBMSCs. hBMSCs received transfection with the help of RPN2‐expressing vector, before osteoblastic differentiation stimulation. (A, B) qRT‐PCR (A) and WB (B) confirmed an up‐regulated RPN2 level at day 14. (C) Alizarin Red staining and von Kossa staining at the 14th day after stimulation. (D) Determination of ALP activity in every group at the 14th day after stimulation. (E) qRT‐PCR was used to examine the expression of OPN, RUNX2, OCN, ALP and BSP of every group at the 14th day after stimulation. These tests were repeated three times. Differences were assessed by two‐tailed Student's *t*‐test. Error bars represent SD. *P < 0.05 versus induced/negative control (NC) groups.

hBMSCs were first transfected with the RPN2‐overexpressing vector to up‐regulate cellular RPN2 and to assess the contribution of RPN2 overexpression to hBMSC osteoblastic differentiation. RPN2 expression of every group underwent verification with qRT‐PCR and WB (Fig. 3A,B). Excessive RPN2 expression also brought about reinforced matrix mineralization (Fig. 3C). Moreover, ALP function was noticeably reinforced via transfection with RPN2‐overexpressing vector (Fig. 3D). Significant up‐regulation of OPN, RUNX2, OCN, ALP and BSP expressions was shown in cells transfected with RPN2 up‐regulation by qRT‐PCR (Fig. 3E).

**Figure 4:**
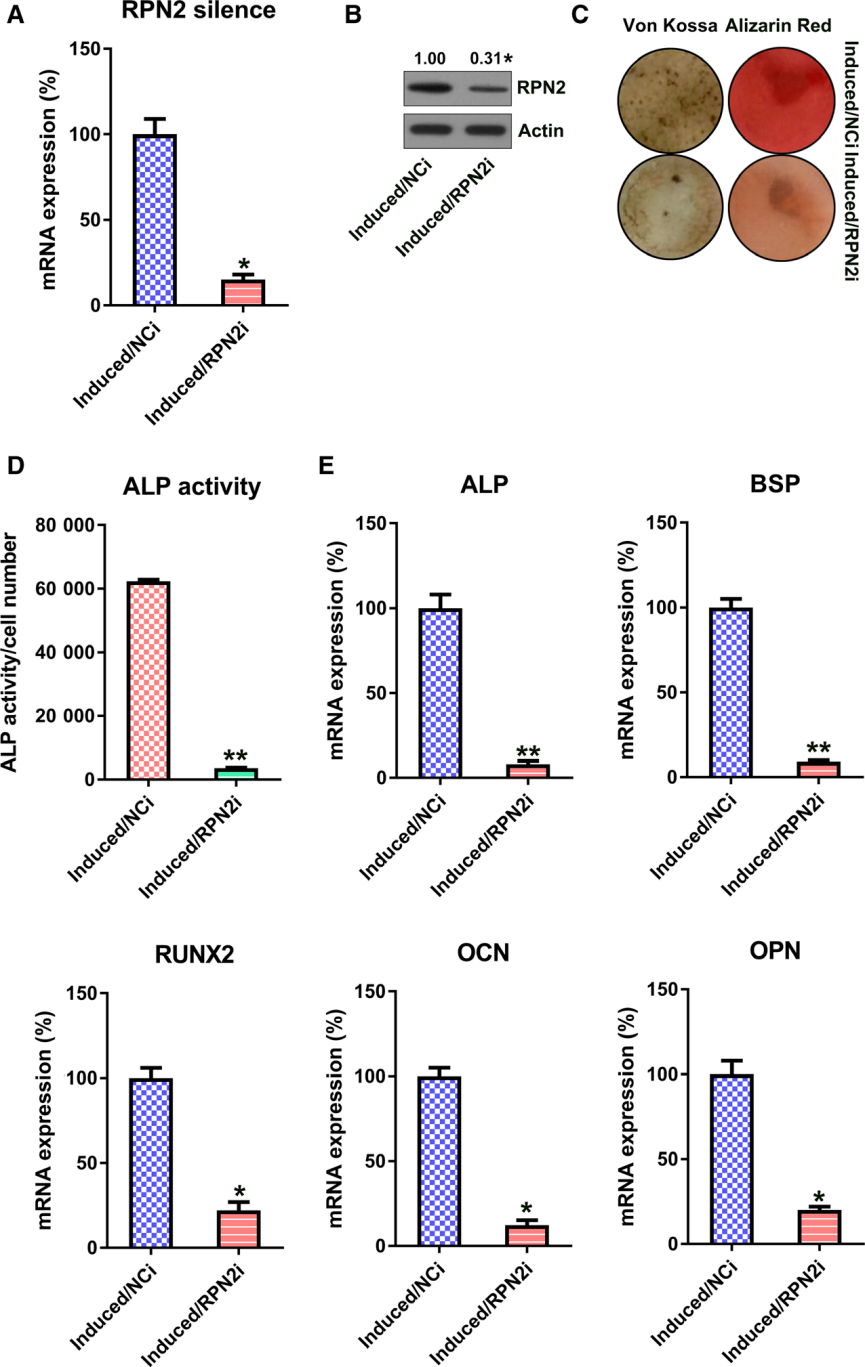
RPN2 silencing inhibited osteoblastic differentiation of hBMSCs. hBMSCs received transfection with the help of RPN2 siRNA or NC siRNA, before induction of osteoblastic differentiation. (A, B) qRT‐PCR (A) and WB (B) confirmed down‐regulated RPN2 level at day 14. (C) Alizarin Red staining and von Kossa staining at the 14th day after stimulation. (D) Determination of ALP activity of every group at the 14th day after stimulation. (E) qRT‐PCR was utilized to examine the expression of OPN, RUNX2, OCN, ALP and BSP of every group at the 14th day after stimulation. These tests were repeated three times. Differences were assessed by two‐tailed Student's *t*‐test. Error bars represent SD. *P < 0.05, **P < 0.01 versus induced/NCi groups. NCi, negative control siRNA.

Then, we attempted to further evaluate the role of RPN2 on osteogenic differentiation of hBMSCs by silencing RPN2 expression. Cells underwent transfection with RPN2 siRNA or negative control (NC) siRNA. Depletion of RPN2 expression was confirmed by qRT‐PCR and WB (Fig. 4A,B). RPN2 silencing also resulted in a decrease in matrix mineralization (Fig. 4C). ALP function was markedly reinforced upon the silencing of RPN2 (Fig. 4D). In addition, ALP, BSP, OCN, OPN and RUNX2 expressions were down‐regulated in induced hBMSCs in the silencing group (Fig. 4E).

### RPN2 down‐regulated the JAK1/STAT3 axis by ubiquitinating JAK1

**Figure 5:**
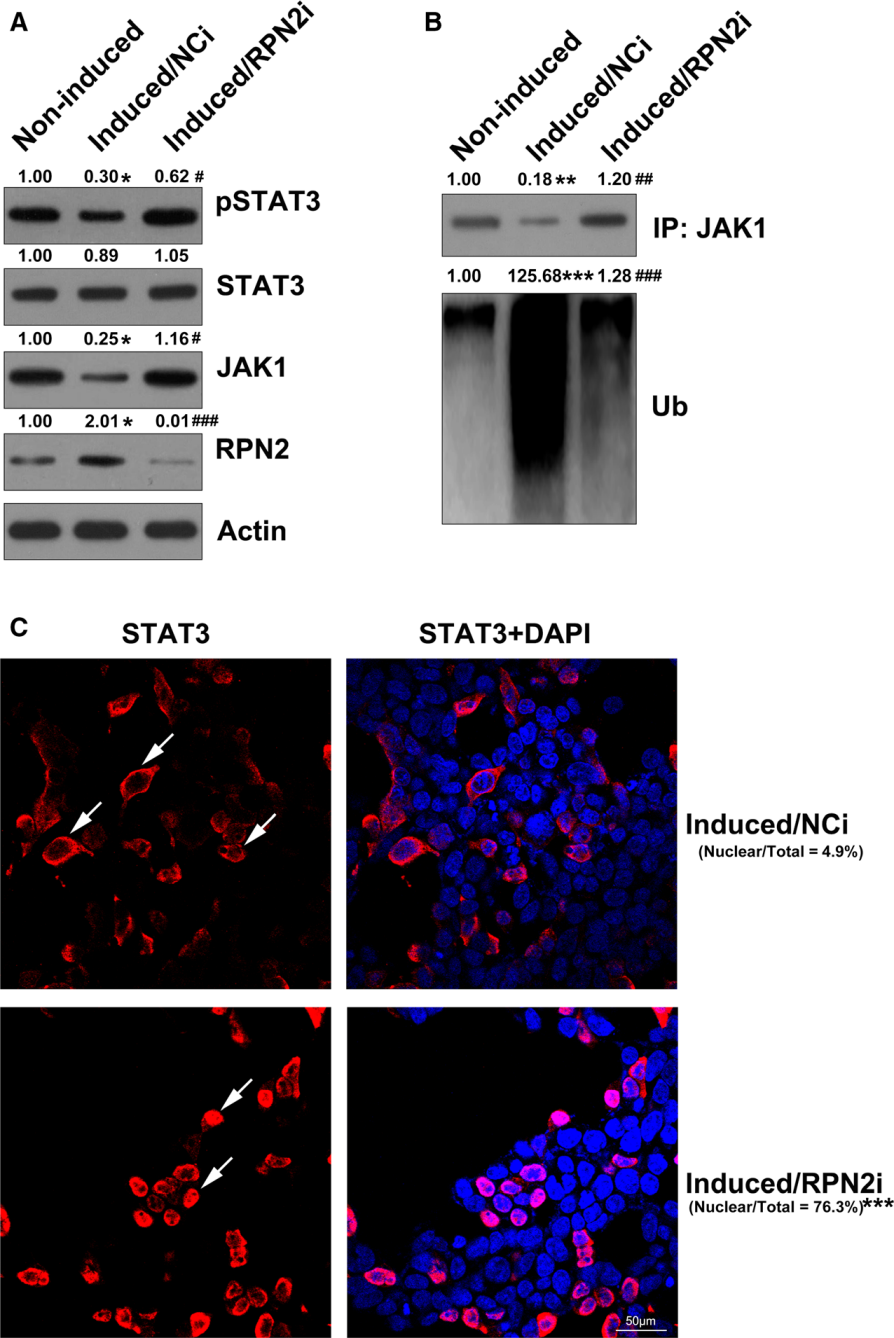
RPN2 regulated the JAK1/STAT3 pathway. hBMSCs received transfection with the help of RPN2 siRNA or NC siRNA before induction of osteoblastic differentiation. (A) WB showed expression of JAK1 and STAT3, and phosphorylation of STAT3 after RPN2 silencing. (B) WB revealed the ubiquitination level of JAK1 after RPN2 silencing. (C) Immunofluorescence assay was used to detect the nuclear location of STAT3. Scale bar, 50 μm. These tests were repeated three times: *P < 0.05, **P < 0.01, ***P < 0.001 versus noninduced groups; ^#^ *P* < 0.05, ^##^ *P* < 0.01, ^###^ *P* < 0.001 versus induced/NCi group. DAPI, 4',6‐diamidine‐2'‐phenylindole dihydrochloride; IP, immunoprecipitation; NCi, negative control siRNA; Ub, ubiquitination.

A previous study has demonstrated that signal transduction of STAT3 and JAK2 is inhibited by RPN2 28. Our research revealed that during hBMSC osteogenic differentiation, expression of JAK1 was markedly reduced; therefore, phosphorylation of its downstream STAT3 was also decreased. However, silencing of RPN2 counteracted their repression, indicating that the JAK1/STAT3 signal axis was activated by RPN2 silencing (Fig. 5A). In addition, it was also found that up‐regulation of RPN2 facilitated the ubiquitination of JAK1, therefore potentially leading to its degradation (Fig. 5B). We also examined the nuclear translocation of STAT3 by immunofluorescence assays, which represented the activation of the JAK/STAT pathway. The results showed that RPN2 up‐regulation also blocked nuclear trafficking of STAT3, but RPN2 silencing contributed to the nuclear location of STAT3 (Fig. 5C). These findings suggested that RPN2 inhibited signal transduction of the JAK/STAT pathway by ubiquitination of JAK1.

### JAK1 contributed to RPN2‐modulated osteogenic differentiation of hBMSCs

**Figure 6:**
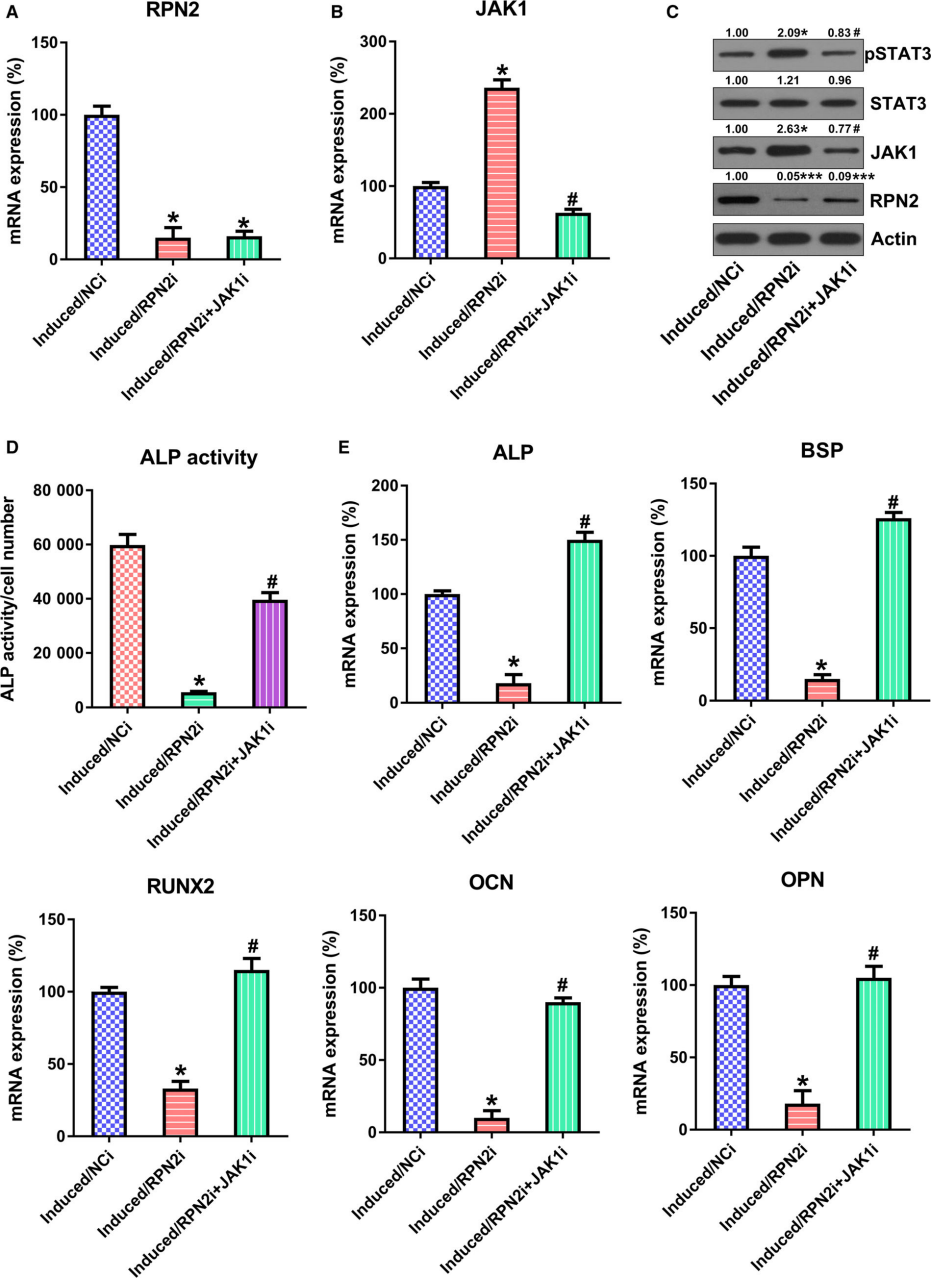
Assessment of the impact of JAK1 on osteoblastic differentiation modulated via RPN2. hBMSCs were cotransfected with RPN2 siRNA and/or JAK1 siRNA before stimulation of osteoblastic differentiation. (A–C) qRT‐PCR (A, B) and WB (C) were conducted to detect protein levels of RPN2 and JAK1. (D) ALP function was examined in every group during the osteoblastic differentiation. (E) qRT‐PCR was performed to examine the osteoblastic markers (OCN, OPN, RUNX2, BSP and ALP) of every group on day 14 after stimulation. These tests were repeated three times. Differences were assessed by ANOVA test. Error bars represent SD. *P < 0.05 versus induced/NCi group; ***P < 0.001; ^#^ *P* < 0.05 versus induced/RPN2i group. NCi, negative control siRNA.

To better investigate the contribution of JAK1 to RPN2‐mediated osteogenic differentiation of hBMSCs, we cotransfected cells with RPN2 siRNA and/or JAK1 siRNA. RPN2, JAK1 and STAT3 phosphorylation levels were significantly reduced after transfection of siRNA, whereas the RPN2 level was not affected by the transfection of JAK1 siRNA at both protein and mRNA levels (Fig. 6A–C). We further observed an increase in ALP activity during osteogenic differentiation of hBMSCs in the JAK1 siRNA transfection group (Fig. 6D). In addition, we detected the production of mRNA associated with osteoblastic differentiation. Expression of the mRNAs was remarkably promoted in hBMSCs cotransfected with RPN2 siRNA and JAK1 siRNA (Fig. 6E).

## Discussion

A previous study demonstrated that, in colorectal cancer, phosphorylation levels of STAT3 and JAK2 were inhibited by RPN2 siRNA 28, indicating that the JAK/STAT pathway was positively correlated with RPN2 expression. The results of this study indicate that RPN2 is a novel promoter of osteogenic differentiation of hBMSCs, which confirms that with JAK1 and STAT3 pathways as a target, overexpression of RPN2 can increase ubiquitination of JAK1 and lead to blockage of the JAK1/STAT3 pathway. Furthermore, upon overexpression and silencing of RPN2 via transfection, ALP activity was increased, whereas expression of relative genes controlling osteogenic differentiation was down‐regulated. However, silencing of JAK1 counteracted this phenomenon, indicating that RPN2 prohibited osteoblast differentiation of hBMSCs partly via inactivating and reducing JAK1/STAT3 through ubiquitination. Our findings also offer an innovative aim for therapy of senile osteoporosis and evidence to prompt additional investigation of the interaction between RPN2 and the JAK1/STAT3 pathway in hBMSC osteogenic differentiation.

The role of JAK1 and STAT3 in the osteogenic differentiation process has not been fully elucidated. JAK1 stimulation has an impact on cell death, migration, invasion and proliferation, and STAT3 is also intimately related to pathways of various malignancies 29, 30. Only a few studies have reported involvement of the JAK1/STAT3 pathway in osteogenic differentiation. Wu *et al*. 31 examined the contribution of cadmium chloride (CdCl_2_) to BMSC osteogenic differentiation. CdCl_2_ influenced BMSC survival and the cytoskeleton, depending on treatment concentration. Microarray evidence indicated that the JAK‐STAT axis contributed to the influence of CdCl_2_ 31. Bioinformatics analysis revealed that some pathways could be related to the osteogenic differentiation abilities of BMSCs, including the JAK/STAT axis 32. Roberts *et al*. 33 discovered that proliferation of human allogeneic serum (hAS) was reinforced with an obvious commitment to osteogenic lineage, as evidenced by Runx2 up‐regulation, ALP function and matrix mineralization. JAK/STAT expression was elevated during the period after pathway assessment. Phosphorylation of STAT3 was also reinforced in hAS‐cultivated human periosteum‐derived cells, of which the repression counteracted the proliferative impact of hAS 33. In our study, the coimmunoprecipitation suggested that RPN2 up‐regulation led to ubiquitination of JAK1, whereas silencing RPN2 reduced the ubiquitinated pattern of JAK1. Furthermore, RPN2 silencing also led to phosphorylation and nuclear location of STAT3, meaning that RPN2 silencing activated the signal transduction of the JAK1/STAT3 axis. The ubiquitination of JAK1, or JAK1 degradation, subsequently deactivated the JAK1/STAT3 pathway. The further “loss‐of‐function” assay used JAK1 silencing in induced hBMSCs, and JAK1 silencing caused promoted osteogenic differentiation of hBMSCs. The blockage of JAK1/STAT3 signal transduction results in the elevation of ALP activity and relative gene expression, indicating an inhibitory role of JAK1/STAT3 on osteogenic differentiation.

However, this study still had several flaws. First, *in vivo* modeling was insufficient to further confirm the role of RPN2 on osteogenic differentiation. Second, a detailed mechanism about the RPN2‐mediated JAK1/STAT3 pathway needs to be fully investigated. To further understand the clinical application of both RPN2 and JAK1/STAT3 pathways, it is necessary to investigate the effects of their inhibitor or inducer on the differentiation and maturation of both osteoblasts and osteoclasts *in vivo* models.

## Conclusions

This study suggests that RPN2 potentially served as a positive modulator of osteogenic differentiation of hBMSCs. Overexpression of RPN2 reinforced the osteogenic differentiation, whereas RPN2 silencing of hBMSCs is recognized as intimately related to down‐regulation of certain osteogenesis‐linked genes, matrix mineralization and ALP function. This indicates that RPN2 could act as a potential marker of osteogenic differentiation. Moreover, the RPN2/JAK1/STAT3 axis could be a potential therapeutic target in other illnesses because of its known effect on inflammatory reactions.

## Conflict of interest

The authors declare no conflict of interest.

## Author contributions

LN and YZ conceived the study and designed the experiments. JY, XG and ZL contributed to the data collection, and XW and HG performed the data analysis and interpreted the results. LN wrote the manuscript. YZ contributed to the critical revision of the article. All authors read and approved the final manuscript.
